# Nicotinamide: A Multifaceted Molecule in Skin Health and Beyond

**DOI:** 10.3390/medicina61020254

**Published:** 2025-02-01

**Authors:** Lara Camillo, Elisa Zavattaro, Paola Savoia

**Affiliations:** Department of Health Science, Università del Piemonte Orientale, 28100 Novara, Italy; lara.camillo@uniupo.it (L.C.); elisa.zavattaro@med.uniupo.it (E.Z.)

**Keywords:** nicotinamide, chemoprevention, skin health, cellular oxidative stress, DNA damage, photoaging, ultraviolet radiation, inflammation, vitamin B3

## Abstract

Nicotinamide (NAM), the amide form of vitamin B3, is a precursor to essential cofactors nicotinamide adenine dinucleotide (NAD⁺) and NADPH. NAD⁺ is integral to numerous cellular processes, including metabolism regulation, ATP production, mitochondrial respiration, reactive oxygen species (ROS) management, DNA repair, cellular senescence, and aging. NAM supplementation has demonstrated efficacy in restoring cellular energy, repairing DNA damage, and inhibiting inflammation by suppressing pro-inflammatory cytokines release. Due to its natural presence in a variety of foods and its excellent safety profile—even at high doses of up to 3 g/day—NAM is extensively used in the chemoprevention of non-melanoma skin cancers and the treatment of dermatological conditions such as blistering diseases, atopic dermatitis, rosacea, and acne vulgaris. Recently, its anti-aging properties have elevated NAM’s prominence in skincare formulations. Beyond DNA repair and energy replenishment, NAM significantly impacts oxidative stress reduction, cell cycle regulation, and apoptosis modulation. Despite these multifaceted benefits, the comprehensive molecular mechanisms underlying NAM’s actions remain not fully elucidated. This review consolidates recent research to shed light on these mechanisms, emphasizing the critical role of NAM in cellular health and its therapeutic potential. By enhancing our understanding, this work underscores the importance of continued exploration into NAM’s applications, aiming to inform future clinical practices and skincare innovations.

## 1. Introduction

Nicotinamide (NAM)—also known as niacinamide—is the amid form of vitamin B3 and is a critical precursor for nicotinamide adenine dinucleotide (NAD^+^) and NADPH. By participating in key oxidation–reduction reactions, NAD⁺ supports cellular homeostasis, metabolism, ATP production, mitochondrial respiration, ROS management, DNA repair, and processes related to aging [1,2,3,4,5]. Studies show that NAM supplementation restores cellular energy, contributes to DNA damage repair, and suppresses inflammation by inhibiting the release of pro-inflammatory cytokines [6]. NAM is naturally present in a variety of foods, including meat, liver, fish, yeast, legumes, nuts, grain products, green leafy vegetables, cereals, coffee, and tea [7,8], making it an essential component of the daily diet. A deficiency of NAM is the primary cause of pellagra, an endemic disease characterized by the triad of dementia, diarrhea, and dermatitis [9].

Unlike other vitamin B3 derivatives (e.g., nicotinic acid, nicotinamide riboside, and nicotinamide mononucleotide), NAM generally shows excellent tolerability—even at doses up to 3 g/day—with fewer adverse effects [10,11,12]. Unlike its derivates, such as nicotinic acid, nicotinamide riboside, and nicotinamide mononucleotide, which may cause side effects like flushing, itching, hypotension, and headaches, NAM is generally safe and well-tolerated, even at high doses (up 3 mg/die), with rare adverse effects observed over prolonged treatments [10,11,12].

Recent research underscores the pivotal role of the skin as the body’s primary interface with external stressors, classifying it as a “stress organ” endowed with complex neuroendocrine capabilities. More than a passive barrier, the skin contains an elaborate network of local endocrine components—including a cutaneous hypothalamic-pituitary-adrenal (HPA)-like axis—enabling it to produce, metabolize, and respond to hormones and neuromediators. These systems collectively protect against various insults, such as pathogens, toxins, and ultraviolet radiation, by modulating inflammatory responses, immune surveillance, and barrier function. For instance, Slominski and colleagues [13] describe how the skin synthesizes and responds to corticotropin-releasing hormone (CRH), adrenocorticotropic hormone (ACTH), cortisol, and other peptides, illustrating the complex local regulatory loops that can operate independently of systemic endocrine pathways. More recent findings [14] further highlight the skin’s sophisticated neuroendocrine network and its ability to orchestrate local immune responses, cell proliferation, and differentiation under stressful conditions. These observations support the concept that the skin is not merely a passive barrier but a dynamic organ adept at sensing and responding to environmental and physiological stressors through interconnected neuroendocrine and immune mechanisms. Owing to its beneficial properties, NAM has found extensive use as a chemopreventive agent for non-melanoma skin cancer (NMSC) and as a treatment for various skin conditions, including blistering diseases, acne vulgaris, and other skin disorders [7]. More recently, NAM has gained popularity as an active ingredient in daily skincare formulations, particularly for its anti-aging effects [15]. Against this background, NAM’s benefits extend beyond DNA repair and energy replenishment to include oxidative stress mitigation, regulation of the cell cycle, and modulation of apoptosis. This review summarizes the molecular mechanism underlying the effects of NAM, focusing on the latest research findings.

## 2. Nicotinamide

### 2.1. General Information and Metabolism

**Figure 1:**
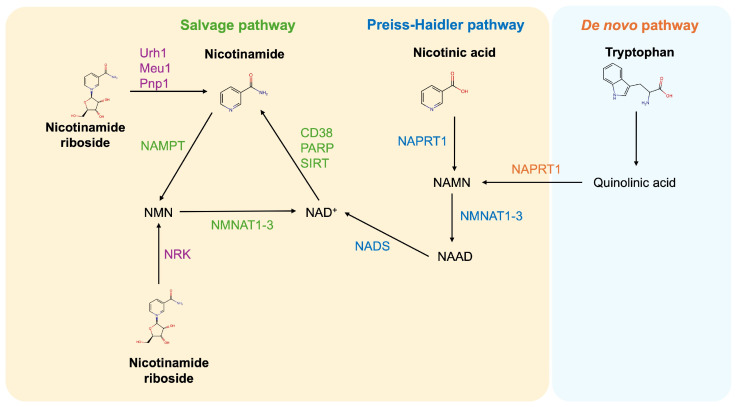
Pathways for NAD^+^ synthesis in mammalian cells. In mammalian cells, NAD^+^ can be synthesized from NA through the Preiss–Haidler pathway or de novo from tryptophan. However, most NAD^+^ is recycled via the salvage pathway from NAM. NAD, nicotinamide adenine dinucleotide; NMN, nicotinamide mononucleotide; NAMN, nicotinic acid mononucleotide; NAAD, nicotinic acid adenine dinucleotide; NADS, NAD synthetase; NMNAT, nicotinamide mononucleotide adenylyl transferase; NAMPT, nicotinamide phosphoribosyl transferase; NAPRT, nicotinic acid phosphorybosiltransferase; Urh1, uridine hydrolase; Meu1, methylthioadenosine phosphorylase; Pnp1, purine nucleoside phosphorylase; NRK, nicotinamide riboside kinase.

NAM is part of the vitamin B3 family, which includes nicotinic acid (NA), nicotinamide mononucleotide (NMN), and nicotinamide riboside (NR) [16]. NAM and NA were first identified in 1937 by Kohen and Elvehjem as the dietary factor able to prevent pellagra symptoms in dogs [17], while NR was discovered in 2004 by Bieganowski and Brenner [18]. All vitamin B3 components are key contributors to NAD^+^ synthesis through distinct metabolic pathways, as illustrated in Figure 1.

The Preiss–Handler pathway converts NA into NAD^+^ via three enzymatic steps: (1) NA is converted to nicotinic acid mononucleotide (NAMN) by nicotinic acid phosphoribosyltransferase (NAPT), (2) NAMN is converted to nicotinic acid adenine dinucleotide (NAAD) by nicotinamide mononucleotide adenylyltransferase (NMNAT), (3) NAAD is converted to NAD^+^ by NAD^+^ synthetase (NADS) [16,19]. The salvage pathway rapidly restores NAD levels by recycling NAM molecules released during enzymatic processes such as SIRT-mediated deacetylation, PARP-mediated ADP-ribosylation, and CD38 activities [20,21,22]. In this pathway, NAM is converted into nicotinamide mononucleotide (NMN) by nicotinamide phosphoribosyl transferase (NAMPT), the rate-limiting enzyme that uses 5′-phosphoribosyl-1-pyrophosphate (PRPP) as a substrate. NAMPT expression is often dysregulated in conditions like obesity, cancers, and metabolic disorders. Its activity is also modulated by circadian rhythms, leading to oscillations in cellular NAD levels [16,23,24,25]. NMN is then converted into NAD^+^ by NMNAT. Finally, NR indirectly supports the salvage pathway through its conversion into NMN by nicotinamide riboside kinase (NRK) or into NAM via uridine hydrolase (Urh1), purine nucleoside phosphorylase (Pnp1), and methylthioadenosine phosphorylase (Meu1) [26,27]. A small proportion of NAD^+^ is synthesized via the de novo pathway which converts tryptophan to NAMN through multiple steps. However, this way is insufficient to maintain and restore NAD^+^ levels (60 mg of tryptophan produces 1 mg of NAD^+^) [16,28].

Beyond its conversion to NAD^+^ through the de novo pathway, tryptophan can also be metabolized in the skin into serotonin and melatonin [29]. The latter, in particular, has garnered attention for its local photoprotective properties, including scavenging of reactive oxygen species and regulation of apoptosis under UV stress [30]. Such processes are highly relevant to skin pathology and photocarcinogenesis, suggesting that localized serotonin–melatonin pathways in the skin could offer both diagnostic and therapeutic insights. Future comparative analyses that integrate these metabolic routes may help clarify the complex interplay between oxidative stress, immune responses, and carcinogenesis in cutaneous tissues.

**Figure 2:**
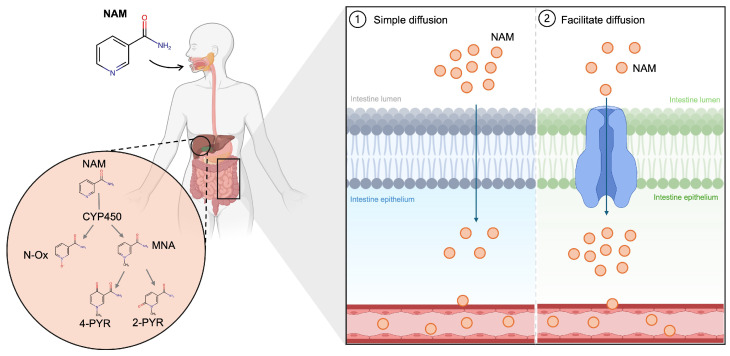
Schematic representation of NAM metabolism. Oral ingested NAM is absorbed in the small intestine through simple or facilitated diffusion, reaching the liver where excess NAM is metabolized by cytochrome P450 NAM into 4-PYR and 2-PYR, which are excreted. NAM, nicotinamide; CYP450, cytochrome P450; N-Ox, nicotinamide N-oxide; MNA, N-methyl-nicotinamide; 4-PYR, N1- methyl-4-pyridone-3-carboxamide; 2-PYR, N1-methyl-2-pyridone-5-carboxamide.

The primary circulating form of vitamin B3 is NAM. NA, predominantly found in plant-based foods, is converted into NAD^+^ in the intestine and liver. NAD^+^ is subsequently cleaved to release NAM into the bloodstream for uptake by extrahepatic tissues [31,32]. Dietary NAM, which is abundant in animal-based foods, is absorbed by the small intestine and transported into endothelial cells through passive diffusion—either simple or facilitated—depending on intracellular NAM concentrations (Figure 2) [33,34]. Excessive NAM is metabolized in hepatocytes by cytochrome P450 into nicotinamide-N-oxide (N-Ox) and N-methyl-nicotinamide (MNA), which is, in turn, converted into N1-methyl-2-pyridone-5-carboxamide (2-pyr) and N1- methyl-4-pyridone-3-carboxamide (4-pyr) [32,35].

Despite their similar chemical structure, NA and NAM differ from a therapeutic point of view. NA is clinically used to manage dyslipidemia due to its ability to bind to the G-protein-coupled receptor 109a (GPCR109a). This binding reduces lipolysis in adipose tissue [36], but it also stimulates the secretion of prostaglandin D2 and E2 by Langerhans cells, leading to side effects such as vasodilation and flushing [37,38]. In contrast, NAM exhibits much greater tolerability, with minimal side effects reported only at high doses (up to 3 g daily) [10,39]. As a result, NAM is extensively utilized in the treatment of various skin disorders, including acne, rosacea, and aging, and as a chemopreventive agent for skin cancers, even in frail patients [32,40,41]. The differences in their side effect profiles may be attributed to NAM’s lack of a carboxyl group in its chemical structure and the absence of specific receptors such as GPCR109A. In Table 1, the differences among vitamin B3 derivates are summarized.

When prescribed *for the treatment of* human skin diseases, the usual oral dosage of NAM is 250–500 mg/*die*, with a good tolerability profile, with nausea and diarrhea only rarely being reported and counteracted by simply taking NAM with food [52]. Some studies have also evaluated its tolerability in special categories such as organ transplant recipients [53] and patients with HIV [54], thus showing the positive effects and safety of NAM. Despite the lack of conclusive data available on the possible genotoxic or carcinogenic effects of NAM [40], its administration at doses higher than 3.5 g daily has been found to be toxic and to potentially cause headache, dizziness, vomiting, and also increase in the liver enzymes and hepatotoxicity [52]. On the contrary, NAM was previously been shown to protect the liver from alcohol [55]. Moreover, high doses of NAM administered intravenously represent the specific therapy to counteract the Vacon poisoning. Vacon is a powder rodenticide able to acutely destroy the pancreatic beta islet cells, thus provoking diabetic ketoacidosis and mental status changes [56]. Luckily, Vacon is not currently available for general use.

The oral administration of NAM allows a transient but significant increase in NAD^+^ blood level, as demonstrated by Ito and colleagues in their clinical trial [57]. Indeed, they have proved that oral supplementation with 500 mg of NAM boosted blood NAD^+^ levels between 12 and 48 h after the administration, finding also blood lipidome change. Other preclinical studies conducted on animal models confirmed the beneficial effects of NAM administration, showing improved lifespan and increased NAD^+^ levels [4,58,59]. Overall, these findings suggest a beneficial role of NAM administration against aging and other diseases due to its ability to restore cellular energy and the absence of side effects with the daily tolerable dose [60].

### 2.2. Mechanisms of Action

#### 2.2.1. DNA Repair

All cells of the human body are continuously exposed to various agents that cause DNA lesions. If these lesions are not repaired or repaired incorrectly, they can eventually lead to mutations [61]. Some DNA damages occur accidentally during DNA replication or can be induced by reactive oxygen species (ROS), which are naturally produced during oxidative respiration [62]. These molecules often cause single-strand breaks (SSBs), characterized by the loss of a single nucleotide and damage at the site of the break [63]. Other DNA damages result from exposure to exogenous agents, such as ultraviolet radiation and chemical or physical factors [64]. To counteract these alterations, cells are equipped with efficient systems, collectively referred to as the DNA damage response (DDR), able to recognize and correct mutated nucleotides [64]. The DDR network comprises numerous proteins and genes that function as DNA lesion sensors and regulate the cell cycle via DNA-damage checkpoints [65]. For example, the nucleotide excision repair (NER) system, which involves PARP-1, addresses lesions typically induced by ultraviolet radiation, while the base excision repair (BER) pathway, including 8-oxoguanine glycosylase (OGG1), targets modifications induced by ROS [66]. To facilitate DNA repair, cells arrest the cell cycle through the activation of p53, which, in turn, inhibits the activity of cyclin-dependent kinases (CDK) until DNA repair is complete [65,67]. However, in case of severe DNA damage, cells may permanently arrest the cell cycle, undergoing apoptosis and senescence [68]. The DDR process requires significant amounts of ATP, leading to rapid consumption of NAD^+^ and a drastic reduction in cellular energy levels. Nevertheless, NAM, as both NAD^+^ precursor and inhibitor of some DDR proteins, can help restore ATP production and promote DNA repair. Indeed, NAM is a specific inhibitor of PARP1 and sirtuin-1 (SIRT1) both part of the DDR system [69]. PARP1 is one of the earliest nuclear proteins recruited to the sites of DNA damage and can repair single- and double-strand DNA breaks by attaching a negatively charged polymer called poly(ADP-ribose) (PAR) to itself and other targets [70]. The ADP units needed for the polymerization of PAR are donated by NAD^+^ [71,72]. Consequently, the prolonged activity of PARP1 induced by persistent stress or severe DNA damage leads to a rapid consumption of nuclear NAD^+^ and causes ATP depletion, critical energy imbalance, and activation of apoptosis [73]. SIRT1 is a NAD-dependent class III histone deacetylase (HDAC III) that regulates cell senescence, apoptosis, cell metabolism, oxidative stress, and inflammation [74,75]. The regulation of SIRT1 expression is controlled at both transcriptional and post-transcriptional levels, including hypermethylation of its specific promoter, p53-induced downregulation, non-coding RNAs (NcRNAs), ubiquitination, glycosylation, and phosphorylation [75,76]. On the other hand, SIRT1 is regulated by additional factors that operate directly on the protein. One of these factors is the NAD^+^/NADH ratio, which correlates with protein activity. Indeed, when NAD^+^ is depleted due to inflammation, oxidative stress, or PARP-1 activity, SIRT1 is downregulated, promoting a chain reaction of inflammation, senescence, and damage [75,77,78]. NAM supplementation transiently inhibits SIRT1, allowing sufficient replenishment of NAD^+^ levels and recovery of the protein [79]. Interestingly, the SIRT1 inhibition mediated by NAM does not affect the deacetylation activity of the protein [40,80]. The ability of NAM to enhance DNA repair is particularly tangible on skin cells, especially after exposure to ultraviolet radiation, as described in Section 3.1.

#### 2.2.2. DNA Damage-Induced Inflammation

Inflammation is an innate response to harmful stimuli and plays a critical role in promoting the healing process [81]. However, unresolved or chronic inflammation, particularly when induced by external factors such as ultraviolet radiation, contributes to the development of chronic diseases, accelerates aging, and plays a role in carcinogenesis [82,83]. In particular, the connection between inflammation and cancer development is complex. Indeed, chronic inflammation originates in a cancer-favoring environment, promoting cellular proliferation and viability, remodeling of extracellular matrix, and favoring cell migration [84]. In particular, the cornerstone of inflammation is the production of ROS designed to counteract pathogens, but it also leads to collateral damage to the host’s DNA [84,85]. The presence of DNA damage induces the activation of the DNA-damage sensor system conducive to the consequences described in Section 2.2.1. On the other hand, the presence of DNA damage is an inducer of inflammation [86]. Indeed, DNA damages induce the expression of type I interferons and other inflammatory factors, which, in turn, contributes to ROS production and DNA mutations [86,87]. NAM supplementation contributes to the reduction of inflammation by enhancing DNA damage repair and indirectly inhibiting the nuclear factor-κB (NF-κB) activity, which is one of the principal factors involved in the inflammatory response [86,88]. Indeed, NF-κB is a family of transcription factors that can be activated through two different pathways: the canonical and non-canonical pathways. The canonical pathway is a rapid and transient activation of NF-κB mediated through Toll-like receptors (TLRs), interleukin-1 β (IL-1β), tumor necrosis factor ɑ (TNFɑ), and lipopolysaccharides (LPS) [89,90,91]. On the other hand, the non-canonical pathway is a slow and persistent activation of NF-κB that is dependent on protein synthesis and triggered by a subset of TNFR signals [92]. This pathway is fundamental for immune system regulation, including B cell maturation and survival, lymphoid organ development, and autoimmune T cell detection [91,92]. Dysregulation of this pathway results in excessive pro-inflammatory cytokines release, leading to autoimmune diseases, chronic inflammation, and contributing to tumor microenvironment [89,93]. Therefore, mitigating inflammation could be an effective strategy to hinder or prevent cancer development [84]. As demonstrated by Torres-Méndez et al. [94] and by Hou et al. [95], NAM supplementation reduced brain inflammation in diabetic mice and transgenic mice models for Alzheimer’s disease, respectively. Also, data presented by Elhassan et al. [96] demonstrated that NR oral supplementation in aged men drastically reduced circulating inflammatory cytokines, together with improved skeletal muscle NAD^+^ metabolome.

#### 2.2.3. Oxidative Stress

The term ROS refers to a group of highly reactive molecules that contain oxygen radicals [97]. ROS are derived from endogenous and exogenous sources. Endogenous ROS are generated as bioproducts of cellular signaling, oxidative respiration, and inflammation processes [98,99]. Exogenous ROS are the consequence of exposure to external stimuli, like ionizing radiation, such as γ- or X-rays, UVA, or oxidizing chemicals [99]. Depending on their concentration and duration of exposure, ROS can have both beneficial and detrimental effects [100]. Nevertheless, in order to keep under control ROS levels, cells are equipped with defense mechanisms, including non-enzymatic ROS scavengers like glutathione (GSH) and vitamin C and E (ɑ-tocopherol), and antioxidant enzymes like superoxide dismutase (SOD), glutathione peroxidase (GPX), and catalase [99,101]. However, when the balance tips in favor of ROS over antioxidants, oxidative stress occurs, leading to significant damage to DNA, protein, and lipid peroxidation [72,102]. Moreover, ROS can somehow activate the mitogen-activated protein kinases/extracellular signal-related kinases (MAPK/ERK) pathways that regulate cell proliferation, differentiation, inflammation, apoptosis, and cell survival [103,104,105]. NAM supplementation has been shown to prevent oxidative stress by reducing ROS levels, thereby protecting cells from their harmful effects [106]. Indeed, Tan et al. [107] have demonstrated that NAM can efficiently reduce oxidative stress induced by UVB radiation in both human primary keratinocytes and three-dimensional organotypic epidermal models, reducing cellular senescence and signs of aging. Similarly, in our recent study, we found that NAM decreases ROS production and oxidative stress in primary keratinocytes isolated from fields of cancerization [108]. Interestingly, NAM protective effects extend beyond the skin. In a mouse model of optical nerve injury, NAM supplementation reduced oxidative stress and neuroinflammation, leading to improved vision [109]. Furthermore, NAM protects natural killer cells from oxidative stress, enhancing their function and remission rates in patients with non-Hodgkin lymphoma [110]. While numerous studies support NAM’s antioxidant role [111], it remains unclear whether its effects are strictly NAD-dependent or if NAM interacts with other pathways or antioxidant molecules. Further research is needed to elucidate these mechanisms.

#### 2.2.4. Aging

Aging is a progressive degenerative state that involves all organs and tissues of the body [112]. It results from a complex interplay of different biological processes, including the accumulation of DNA damage, telomers shortening, NAD^+^ depletion, chronic inflammation, and cellular senescence, all of which contribute to functional decline [112]. These processes are interconnected and can be triggered by both endogenous and exogenous stimuli, like UV, chemicals, and chemotherapy [113]. Among these factors, DNA damage plays a central role in driving aging [67]. Indeed, the activation of the DDR system in response to DNA damages triggers several pathways that ultimately converge on p53 activation, which, in turn, promotes the transcription of cyclin-dependent kinase inhibitors p16 and p21, leading to permanent cell cycle arrest—a state known as senescence [114,115]. Senescence primarily occurs in proliferating cells exposed to persistent stimuli. In addition to the cell cycle, senescent cells exhibit several detrimental characteristics. They overexpress anti-apoptotic enzymes, secrete senescence-associated secretory phenotypes (SASP) that include pro-inflammatory cytokines, growth factors, and proteases, and display increased oxidative stress [116,117,118]. Collectively, these changes, along with the accumulation of senescent cells, compromise tissue integrity and actively contribute to the aging process [116,117,118,119]. Moreover, SASP-induced inflammation and DDR activation are highly energy-demanding processes, further depleting NAD^+^ levels, which naturally decline during aging [5,120]. Compounding these issues, senescent cells appear to exhibit impaired NAMPT activity, although it is not clearly understood [5]. One potential strategy to mitigate aging is the supplementation of NAD^+^ precursors, such as NMN, NR, or NAM, in order to restore NAD^+^ levels and inhibit highly energy-consuming enzymes. As demonstrated by Kang et al. [121] and Mahajan et al. [122], NAM-treated human primary fibroblasts presented reduced aging markers expression, attenuated ROS production, decreased senescence, and restored cell cycle. Also, Oblong et al. [123] have proved that fibroblasts isolated from aged donors, when stimulated with NAM, present improved mitochondrial functions and bioenergetic availability.

## 3. Nicotinamide and Skin

### 3.1. NAM and UV-Induced Effects

**Figure 3:**
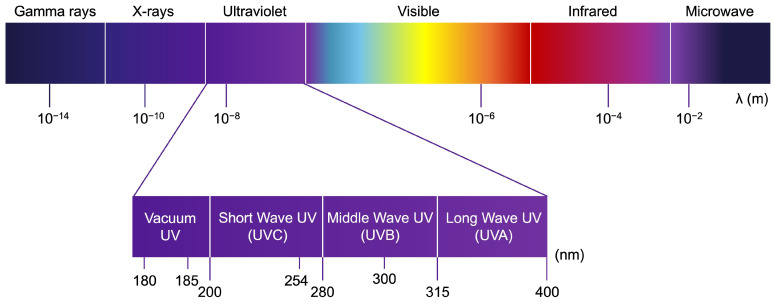
Representation of the electromagnetic spectrum emitted by the sun. UVC is known for its germicidal properties since it can seriously damage RNA and DNA of bacteria and fungi [125]. UVB catalyzes the reactions of vitamin D synthesis, in particular between 290 and 315 nm (280 nm wavelength is absorbed by the atmosphere), but is also responsible for acute and chronic effects, including skin carcinogenesis [126,127]. UVA is widely used in clinics for phototherapy but also accelerates skin aging and indirectly contributes to cancer development [128].

Ultraviolet radiation (UVR) is the main threat to skin integrity. UVR encompasses three types of radiation: UVC (200–280 nm), UVB (280–315 nm), and UVA (315–400 nm) (Figure 3). The ozone layer effectively blocks UVC and the majority of UVB radiation (approximately 90–95%) emitted by the sun. As a result, the UV radiation that reaches the Earth’s surface is predominantly composed of UVA with a small percentage of UVB (5–10%), depending on factors such as latitude, ozone layer thickness, and geographic location [124].

UVR exposure is fundamental for vitamin D production [129], but it also exerts a range of other beneficial effects. Emerging evidence reveals that UVR can induce both local and systemic responses, including modulation of immune function and regulation of neuroendocrine pathways, independent of its role in vitamin D production. [130].

However, both UVA and UVB are responsible for dreaded acute or chronic effects on the skin [131,132]. Acute effects encompass sunburn, tanning, local immunosuppression, and photodamage [133], while chronic effects include photocarcinogenesis and photoaging [132,134]. UV photodamages are caused by both direct and indirect actions on several cellular structures. Indeed, UVB photons are directly absorbed by the DNA, causing the formation of cyclobutane-pyrimidine dimers (CPDs) and pyrimidine-pyrimidone (6-4) photoproducts ((6-4)-PP), leading to mutations [135]. On the other hand, UVA triggers the production of ROS and reactive nitrogen species (RNS), which, in turn, contribute to DNA damage, lipid and protein peroxidation, and the activation of the inflammatory response [135,136,137,138]. However, even if these mechanisms are generally demarcated, some overlaps do exist [139].

So far, several photoprotective strategies have been adopted to mitigate or prevent these UV photodamages, which include the application of sunscreens, wearing photoprotective clothes, and the use of chemopreventive molecules like nicotinamide [140,141]. In humans, NAM has been shown to counteract UV-induced damage through its immune-protective properties and its involvement in energy-dependent cellular processes, including post-irradiation DNA repair. A 2009 study by Yiasemides et al. [142] investigated these effects in six healthy volunteers who received a single sub-erythemal dose of solar-simulated UV (ssUV) radiation on the lower back. Participants were then treated with either 5% nicotinamide lotion or a placebo. A microarray analysis of skin biopsies revealed that UV exposure caused downregulation of genes associated with energy metabolism, immune response, and anti-apoptotic pathways in placebo-treated skin. In contrast, these changes were not observed in skin treated with nicotinamide, highlighting its protective and restorative effects at the molecular level. Moreover, two independent studies [142,143] demonstrated that nicotinamide significantly mitigates solar-simulated UV (ssUV)-induced suppression of Mantoux reactions. This effect was observed in volunteers who received either oral nicotinamide at a dose of 500 mg daily or topical 5% nicotinamide, underscoring its ability to preserve immune function following UV exposure. To confirm these results, in vitro studies have been conducted on skin cells also to unravel the molecular mechanism behind NAM’s photoprotective effects. Indeed, NAM can efficiently reduce aging markers, oxidative stress, and senescence, restore cell cycle arrest, enhance DNA repair, and modulate inflammation in UVB-irradiated human fibroblasts [144] and primary keratinocytes [107,108,145,146,147,148]. Similar effects have been found by Chhabra et al. [149] on UVA/UVB-irradiated normal melanocytes treated with NAM. The possible ways through which NAM can exert its protective effects are the restoration of NAD^+^ levels within cells and the inhibition of NAD-dependent enzymes, facilitating replenishment of cellular energy necessary for the DNA-damage repair system and all cellular reactions. Nevertheless, so far, this hypothesis is still not totally confirmed due to the scarcity of data available in the literature. Therefore, further studies are fundamental to unveil all NAM’s interactions within cells in order to reinforce the importance of this molecule for the treatment and prevention of several skin disorders, including cancers.

Currently, numerous studies are underway exploring the potential applications of nicotinamide in preventing and treating not only skin cancer but also a variety of other dermatological conditions. The most relevant studies are summarized in Table 2.

### 3.2. Photoaging

Unlike other organs, skin aging is accelerated by the action of external stimuli, such as ultraviolet exposure, leading to premature aging or photoaging [150]. The main consequences of photoaging are irregular pigmentation, roughness, telangiectasias, deep wrinkles, and precancerous lesions [151]. Therefore, photoaging treatment and prevention is essential to reduce the risk of developing these modifications [152]. Clinical studies have demonstrated that topical nicotinamide provides a wide range of benefits for improving the appearance of aging facial skin. However, questions regarding its long-term efficacy and safety remain, highlighting the need for further research. A study by Bisset et al. [153] supports these findings. In their 12-week, double-blind, placebo-controlled, split-face trial, 50 Caucasian women aged 40 to 60 years were randomly assigned to use a control moisturizer on one side of the face and the same moisturizer containing 5% nicotinamide on the other. Nicotinamide was well-tolerated and showed significant improvements compared to the control in multiple parameters, including fine lines and wrinkles, hyperpigmentation spots, skin texture, and red blotchiness. Notably, it also significantly reduced skin yellowing compared to the control.

Additionally, cosmetic formulations containing nicotinamide have been shown to enhance the stratum corneum’s aqueous content and reduce transepidermal water loss, resulting in improved skin appearance, firmness, elasticity, and reduced wrinkles [154]. The anti-aging effectiveness of nicotinamide was further confirmed in a study by Bogdanowicz et al. [155], which documented improvements in fine lines, wrinkles, luminosity, smoothness, homogeneity, and plumpness in 44 women treated with a topical product containing both nicotinamide and hyaluronic acid. These effects were accompanied by a decrease in the expression of several senescence-associated secretory phenotype (SASP) genes.

### 3.3. Non-Melanoma Skin Cancer

Recent clinical studies have provided compelling evidence supporting the role of nicotinamide in skin cancer prevention. In 2015, Kim et al. [12] presented the combined findings of two randomized, double-blind phase 2 trials involving 74 participants with photo-damaged skin and a history of skin cancer. These trials evaluated the effect of oral nicotinamide on actinic keratosis (AK) counts and demonstrated a significant reduction in the likelihood of developing at least one skin cancer among participants treated with nicotinamide compared to those receiving a placebo. Similarly, two Australian phase 2, double-blind, randomized, placebo-controlled trials reported that a daily dose of 500 mg oral nicotinamide led to a reduction in precancerous lesions and in situ skin carcinomas after just four months of treatment [8]. A landmark randomized trial published by Chen et al. in 2015 [39] further underscored these findings, showing that oral nicotinamide significantly reduced the incidence of new non-melanoma skin cancers and actinic keratoses in high-risk individuals, including kidney transplant recipients. Drago et al. [53] conducted a focused investigation into the effects of 500 mg daily nicotinamide on AKs in solid organ transplant recipients. Thirty-eight patients were randomized to receive either nicotinamide or placebo. Among those treated with nicotinamide, 88% experienced a reduction in AK size, 42% showed complete regression of AKs, and no new AKs developed during the study period. In contrast, 91% of the control group experienced an increase in the number or size of AKs, with seven lesions progressing to squamous cell carcinoma (SCC). Importantly, no skin cancers were reported in the nicotinamide group, highlighting its protective potential against both precancerous and malignant lesions. These findings collectively demonstrate nicotinamide’s efficacy in reducing the burden of skin cancer and precancerous lesions, particularly in high-risk populations. However, another randomized controlled trial conducted in 2023 [156] that enrolled 154 solid organ transplant recipients with a previous diagnosis of skin cancer failed to find differences in the rates of subsequent cancer development between patients treated with nicotinamide and the placebo group. Regarding topical treatment, a randomized clinical trial by de Castro [157] evaluated the effectiveness of 5% nicotinamide cream in maintaining the clinical response achieved after cryosurgery for actinic keratoses (AKs). The study confirmed the safety and tolerability of the product; however, no significant differences in AK reduction or complete clearance were observed between the treatment and placebo groups.

In our experience [158], a topical formulation containing high-protection sunscreens, a DNA Repair Complex with antioxidant and reparative properties targeting UV-induced DNA damage, and nicotinamide demonstrated efficacy in managing AKs in both immunosuppressed and immunocompetent patients. The reduction in AKASI scores ranged from 22.76% to 31.73%, respectively, highlighting its potential as an adjunctive therapy.

## 4. Other Skin Disorders

### 4.1. Acne

The role of nicotinamide in the treatment of acne is well established and was already proposed in the mid-90s [159] and confirmed in subsequent randomized studies [160,161,162] as a potential alternative to topical antibiotics. The proposed mechanism involves the downregulation of matrix metalloproteinases, such as MMP-1, MMP-2, MMP-9, and MMP-14, which play a role in acne pathogenesis, as well as the modulation of pro-inflammatory cytokines [163]. Additionally, nicotinamide has been shown to reduce sebaceous gland activity. While the exact mechanism has not yet been fully elucidated, it has been hypothesized that nicotinamide is converted into niacin, a B-group vitamin; this molecule, in turn, can interact with the HCA2 receptor, raise calcium levels and consequently reduce sebum production [15]. Nicotinamide has also been suggested as a part of topical combination therapies alongside other molecules with keratolytic, antiseptic, and antimicrobial activity [163,164,165]. A randomized study [166] demonstrated a synergic effect when nicotinamide was combined with benzoyl peroxide. In addition, the oral administration of nicotinamide as a part of dietary supplements has been proposed. Two open-label, prospective studies involving 198 and 235 patients, respectively, affected by inflammatory acne [159,167] reported a visible amelioration in most patients, with no significative differences compared to those receiving concomitant antibiotic treatments. More recently, nicotinamide, in the form of niacinamide, was also proposed as a concomitant topical treatment able to reduce the cutaneous side effects (i.e., redness, dryness, and itching) in patients treated with oral isotretinoin for severe acne [168].

### 4.2. Bullous Diseases

Several clinical cases published in the late 20th century [169,170,171,172,173,174] explored with conflicting results the use of nicotinamide in treating patients suffering from different autoimmune bullous diseases, including acquired epidermolysis bullosa. Among these, only the case reported by Honl [169] demonstrated a clinical response to nicotinamide as monotherapy. In all other cases, the anti-inflammatory effect of tetracyclines and the immunomodulatory action of nicotinamide was exploited synergistically. In 2000, an open trial involving 11 patients with bullous pemphigoid treated with tetracycline and nicotinamide (1.5–2 g/daily) showed promising results: six patients achieved an almost complete response, and two others demonstrated a partial response [175]. More recently, in 2019, Kalinska-Bienias [176] published data from a 3-year retrospective study that included 106 patients with bullous pemphigoid. This study compared the efficacy of a treatment regimen combining tetracycline, nicotinamide, and topical steroids with prednisone monotherapy. The combination therapy demonstrated comparable efficacy in disease control while offering improved 1-year and 3-year survival rates for patients treated with the nicotinamide combination. Several clinical cases published in the late 20th century [169,170,171,172,173] explored the use of nicotinamide in treating patients suffering from different autoimmune bullous diseases. Among these, only the case reported by Honl [169] demonstrated a clinical response to nicotinamide as monotherapy. In all other cases, the anti-inflammatory effect of tetracyclines and the immunomodulatory action of nicotinamide was exploited synergistically. In 2000, an open trial involving 11 patients with bullous pemphigoid treated with tetracycline and nicotinamide (1.5–2 g/daily) showed promising results: six patients achieved an almost complete response, and two others demonstrated a partial response [175]. More recently, in 2019, Kalinska-Bienias [176] published data from a 3-year retrospective study that included 106 patients with bullous pemphigoid. This study compared the efficacy of a treatment regimen combining tetracycline, nicotinamide, and topical steroids with prednisone monotherapy. The combination therapy demonstrated comparable efficacy in disease control while offering improved 1-year and 3-year survival rates for patients treated with the nicotinamide combination.

### 4.3. Atopic Dermatitis

The essential role of nicotinamide in managing atopic dermatitis is supported by numerous studies. Notably, El-Heis et al. in 2016 [177] observed that higher concentrations of nicotinamide in maternal serum were associated with a reduced risk of developing atopic dermatitis at 12 months of age. Also, the antipruritic effect of nicotinamide and its role in restoring the skin barrier, likely mediated through its interaction with AQP3, which is overexpressed in atopic dermatitis, makes this molecule mainly effective in treating this pathological condition [42,178,179]. In 2005, Soma et al. [180] conducted a study on 28 atopic patients treated with 2% nicotinamide cream for 4–8 weeks. They observed a significant reduction in the transepidermal water loss (TEWL) and an increase in stratum corneum hydration compared to untreated areas or areas treated with white petrolatum in the same patient. More recently, Zhu et al. [181] demonstrated the efficacy of a nicotinamide-containing cleansing gel and body emollient in improving clinical symptoms and quality of life in patients affected by mild AD. Among 84 patients treated with nicotinamide-based topical products, significant reductions were observed in the SCORAD clinical score, as well as in POEMS and DLQI questionnaire results, compared to the control group. Additionally, substantial improvements in TEWL and stratum corneum water content measurement were documented, indicating skin barrier restoration.

Nicotinamide also shows a synergistic effect when combined with topical tacrolimus, allowing for a reduction in the required dose of tacrolimus for effective AD treatment [182].

### 4.4. Others

Nicotinamide has shown efficacy in managing other various dermatological conditions, including rosacea, psoriasis, and melasma. In rosacea, a common facial dermatosis characterized by flares triggered by exposome factors, topical application of 0.25% N-methylnicotinamide gel—a metabolite of nicotinamide—achieved improvement in 76% of treated patients [183]. Additional topical formulations containing nicotinamide have also demonstrated efficacy in alleviating symptoms in rosacea patients [184,185,186] also in randomized trials [187]. The NICOS (Nicomide Improvement in Clinical Outcomes) study evaluated oral nicotinamide combined with copper, zinc, and folic acid in patients with rosacea and acne vulgaris, reporting moderate to substantial improvement in 79% of participants after four weeks [167].

In psoriasis, a 2010 randomized, double-blind, placebo-controlled study by Levine et al. [188] assessed the efficacy of a combination of calcipotriene and nicotinamide. Although the results were not statistically significant, patients receiving nicotinamide showed a dose-dependent trend of clinical improvement. More recently, El-Khalawani et al. [189] demonstrated that a 4% topical nicotinamide formulation applied twice daily for 12 weeks yielded satisfactory short-term results in 60 patients with mild-to-moderate psoriasis. This benefit may be attributed to nicotinamide’s capacity to suppress dendritic cell activation, as shown in vitro [190].

Topical formulations containing 2% to 5% nicotinamide have demonstrated efficacy in treating melasma and other forms of UV-induced hyperpigmentation. Melasma is characterized by the appearance of brownish or grayish patches on sun-exposed areas of the skin, driven by a combination of genetic, hormonal, and environmental factors. Ultraviolet (UV) radiation can also induce other hyperpigmentation conditions—such as freckles, solar lentigines, and post-inflammatory hyperpigmentation—by stimulating increased melanin production as a protective response to DNA damage. Beneficial effects have been observed both when nicotinamide is used alone and in combination with N-acetyl glucosamine or cysteamine, highlighting its potential as a therapeutic option for pigmentary disorders [153,191,192]. Moreover, a 2011 clinical trial published by Navarrete-Solis [193] compared 4% nicotinamide cream with hydroquinone, a well-known depigmenting agent. While both treatments led to an improvement, hydroquinone showed a slightly superior efficacy, whereas nicotinamide offered better tolerability. Histopathologic analysis revealed reduced mast cell infiltration and improved solar elastosis in nicotinamide-treated areas. Also, DNA hypermethylation has been shown to be reduced in melasma skin areas after treatment with nicotinamide 4% [194].

Finally, a possible role of nicotinamide can also be hypothesized in the stimulation of re-epithelization after surgical or pathological wounds [195,196]. In fact, this molecule can support wound healing processes primarily by enhancing cellular energy metabolism, moderating inflammation, and promoting healthy skin cell function. Also, a possible role in preventing fibrotic scarring has been hypothesized [197].

These findings highlight the versatility of nicotinamide as a therapeutic agent in dermatology, offering anti-inflammatory, immunomodulatory, and skin barrier-enhancing benefits across a spectrum of skin conditions.

## 5. Conclusions

In conclusion, nicotinamide emerges as a versatile, well-tolerated, and clinically relevant molecule with broad applications in dermatology and beyond. As a NAD^+^ precursor, it supports multiple cellular functions that are central to maintaining genomic stability and metabolic homeostasis. By facilitating DNA repair, modulating inflammatory pathways, and attenuating oxidative stress, nicotinamide not only assists in preventing cellular damage but also counters processes that drive aging and carcinogenesis. In the skin, its robust profile is evident: from photoaging and non-melanoma skin cancer prevention to the management of acne, atopic dermatitis, and other chronic inflammatory conditions, nicotinamide exhibits preventive, reparative, and protective properties that improve both clinical outcomes and patient quality of life. Although its safety profile is well-established even at high doses, questions remain regarding the precise mechanisms through which it exerts its beneficial effects—whether entirely through NAD-dependent pathways or via other yet-to-be-clarified molecular interactions. Further research is warranted to fully elucidate the long-term safety and efficacy of nicotinamide supplementation, to optimize dosing strategies, and to identify potential synergistic formulations. Besides, the identification of new biomarkers linked with the efficacy of NAM treatment will allow a better classification of patients that can take advantage of NAM administration and predict its positive response. As our understanding deepens, nicotinamide may become a key pillar in integrated therapeutic approaches for a range of skin conditions and systemic diseases, bridging the gap between nutritional supplementation, pharmacological intervention, and preventive healthcare strategies.

## Figures and Tables

**Table 1:** Overview of the differences among vitamin B3 derivates. ND, no detected.

Vitamin B3 Derivate	Molecular Mechanism	Clinical Use	Side Effects
Nicotinamide (NAM)	- Restore NAD^+^ - DNA damage repair - Immunosuppression - Reduction of ROS - Inhibition of nitric oxide synthase [42,43]	- Pellagra - Chemoprevention - Antimicrobial - Antipruritic - Anti-inflammatory - Treatment of acne, rosacea, melasma, atopic dermatitis [42,44,45]	Visible at high doses (up to 3 mg/die): - Hepatic insufficiency - Nausea - Diarrhea - Flushing [6,46]
Nicotinic acid (NA)	- Reduction of lipolysis in adipose tissue - Stimulation secretion of prostaglandin D2 and E2 - Activation of GPCR109A receptor [47]	- Dyslipidemia - Pellagra [48,49]	- Vasodilation and flushing - Gastrointestinal disorders - Rash - Hyperglycemia - Hyperuricemia [50]
Nicotinamide riboside (NR)	- Restore NAD^+^	Clinical trials [26,51]	ND

NAM Administration and Possible Side Effects.

**Table 2:** List of the most relevant clinical trials on nicotinamide in dermatological diseases.

Schedule/Duration	CT ID	Target	Clinical Phase
NICOTINAMIDE 500 mg/TD + corticosteroids; 12 weeks.	NCT03260166	Cutaneous or systemic lupus erythematosus	Phase II
NICOTINAMIDE 2% or 4% topical; 12 weeks.	NCT05362188	Cutaneous lupus erythematosus	Phase I
NICOTINAMIDE 500 mg/TD vs placebo; 4 weeks.	NCT04271735	Psoriasis	NA
NICOTINAMIDE 4% topical + calcipotriol 0.005%; 12 weeks.	NCT01763424	Psoriasis	Phase II/III
NICOTINAMIDE topical vs. calcipotriol vs. DPS-012 vs. placebo; 12 weeks.	NCT01368887	Psoriasis (scalp)	Phase II
NICOTINAMIDE 4% topical vs. virgin coconut oil; 4 weeks.	NCT04218500	Occupational hand dermatitis	NA
NICOTINAMIDE cosmetic product vs. placebo; 1 week.	NCT06331390	Irritant contact dermatitis	NA
NICOTINAMIDE cosmetic product; 4 weeks.	NCT05454722	Atopic dermatitis	NA
NICOTINAMIDE topical + adapalene; 6 weeks.	NCT03626298	Acne vulgaris	Phase IV
NICOTINAMIDE 2% topical + 0.5% zync + ac. hyaluronic; 8 weeks.	NCT06120452	Acne vulgaris and post-acne hyperpigmentation	NA
NICOTINAMIDE 4% topical vs. placebo; 4 weeks.	NCT01542138	Axillary hyperpigmentation	Phase IV
NICOTINAMIDE 2% topical vs. metformin 30%; 9 weeks.	NCT05790577	Melasma	Phase II
NICOTINAMIDE topical + arbutin + plant extracts; 20 weeks	NCT05986123	Post-inflammatory Hyperpigmentation	NA
NICOTINAMIDE cosmetic product vs. others; 12 weeks.	NCT06770127	Facial post-inflammatory Hyperpigmentation	Observational
NICOTINAMIDE 500 mg/TD vs placebo; 52 weeks	NCT03769285	NMSC prevention (Transplanted patients)	Phase II
NICOTINAMIDE 500 mg/TD 52 weeks	NCT04843553	AK prevention (Transplanted patients)	Phase I

NA: Not applicable.

## Data Availability

Not applicable.

## References

[1] Lushchak O., Gospodaryov D., Strilbytska O., Bayliak M., Çakatay U., Atayik M.C. (2023). Chapter Five-Changing ROS, NAD and AMP: A path to longevity via mitochondrial therapeutics. Advances in Protein Chemistry and Structural Biology.

[2] Munk S.H.N., Merchut-Maya J.M., Rubio A.A., Hall A., Pappas G., Milletti G., Lee M., Johnsen L.G., Guldberg P., Bartek J. (2023). NAD+ regulates nucleotide metabolism and genomic DNA replication. Nat. Cell Biol..

[3] Zapata-Pérez R., Wanders R.J.A., van Karnebeek C.D.M., Houtkooper R.H. (2021). NAD ^+^ homeostasis in human health and disease. EMBO Mol. Med..

[4] Covarrubias A.J., Perrone R., Grozio A., Verdin E. (2020). NAD+ metabolism and its roles in cellular processes during ageing. Nat. Rev. Mol. Cell Biol..

[5] Chini C.C.S., Cordeiro H.S., Tran N.L.K., Chini E.N. (2023). NAD metabolism: Role in senescence regulation and aging. Aging Cell.

[6] Snaidr V.A., Damian D.L., Halliday G.M. (2019). Nicotinamide for photoprotection and skin cancer chemoprevention: A review of efficacy and safety. Exp. Dermatol..

[7] Forbat E., Al-Niaimi F., Ali F.R. (2017). Use of nicotinamide in dermatology. Clin. Exp. Dermatol..

[8] Surjana D., Halliday G.M., Martin A.J., Moloney F.J., Damian D.L. (2012). Oral Nicotinamide Reduces Actinic Keratoses in Phase II Double-Blinded Randomized Controlled Trials. J. Investig. Dermatol..

[9] Karthikeyan K., Thappa D.M. (2002). Pellagra and skin. Int. J. Dermatol..

[10] Knip M., Douek I.F., Moore W.P.T., Gillmor H.A., McLean A.E.M., Bingley P.J., Gale E.A.M., for the ENDIT Group (2000). Safety of high-dose nicotinamide: A review. Diabetologia.

[11] Chen A.C., Damian D.L. (2014). Nicotinamide and the skin. Australas. J. Dermatol..

[12] Kim B., Halliday G.M., Damian D.L. (2015). Oral nicotinamide and actinic keratosis: A supplement success story. Curr. Probl. Dermatol..

[13] Slominski A.T., Żmijewski M.A., Skobowiat C., Zbytek B., Slominski R.M., Steketee J.D. (2012). Sensing the Environment: Regulation of Local and Global Homeostasis by the Skin’s Neuroendocrine System. Advances in Anatomy, Embryology and Cell Biology.

[14] Slominski A.T., Slominski R.M., Raman C., Chen J.Y., Athar M., Elmets C. (2022). Neuroendocrine Signaling in the Skin with a Special Focus on the Epidermal Neuropeptides. Am. J. Physiol. Cell Physiol..

[15] Marques C., Hadjab F., Porcello A., Lourenço K., Scaletta C., Abdel-Sayed P., Hirt-Burri N., Applegate L.A., Laurent A. (2024). Mechanistic Insights into the Multiple Functions of Niacinamide: Therapeutic Implications and Cosmeceutical Applications in Functional Skincare Products. Antioxidants.

[16] Kirkland J.B., Meyer-Ficca M.L., Eskin N.A.M. (2018). Chapter Three-Niacin. Advances in Food and Nutrition Research.

[17] Koehn C., Elvehjem C. (1937). Further Studies on the Concentration of the Antipellagra Factor. J. Biol. Chem..

[18] Bieganowski P., Brenner C. (2004). Discoveries of Nicotinamide Riboside as a Nutrient and Conserved NRK Genes Establish a Preiss-Handler Independent Route to NAD+ in Fungi and Humans. Cell.

[19] Amjad S., Nisar S., Bhat A.A., Shah A.R., Frenneaux M.P., Fakhro K., Haris M., Reddy R., Patay Z., Baur J. (2021). Role of NAD+ in regulating cellular and metabolic signaling pathways. Mol. Metab..

[20] Warren A., Porter R.M., Reyes-Castro O., Ali M., Marques-Carvalho A., Kim H.-N., Gatrell L.B., Schipani E., Nookaew I., O’brien C.A. (2023). The NAD salvage pathway in mesenchymal cells is indispensable for skeletal development in mice. Nat. Commun..

[21] Kennedy B.E., Sharif T., Martell E., Dai C., Kim Y., Lee P.W., Gujar S.A. (2016). NAD+ salvage pathway in cancer metabolism and therapy. Pharmacol. Res..

[22] Braidy N., Berg J., Clement J., Khorshidi F., Poljak A., Jayasena T., Grant R., Sachdev P. (2019). Role of Nicotinamide Adenine Dinucleotide and Related Precursors as Therapeutic Targets for Age-Related Degenerative Diseases: Rationale, Biochemistry, Pharmacokinetics, and Outcomes. Antioxid. Redox Signal..

[23] Nakahata Y., Sahar S., Astarita G., Kaluzova M., Sassone-Corsi P. (2009). Circadian Control of the NAD ^+^ Salvage Pathway by CLOCK-SIRT1. Science.

[24] Garten A., Schuster S., Penke M., Gorski T., de Giorgis T., Kiess W. (2015). Physiological and pathophysiological roles of NAMPT and NAD metabolism. Nat. Rev. Endocrinol..

[25] Ramsey K.M., Yoshino J., Brace C.S., Abrassart D., Kobayashi Y., Marcheva B., Hong H.-K., Chong J.L., Buhr E.D., Lee C. (2009). Circadian Clock Feedback Cycle Through NAMPT-Mediated NAD ^+^ Biosynthesis. Science.

[26] Damgaard M.V., Treebak J.T. (2023). What is really known about the effects of nicotinamide riboside supplementation in humans. Sci. Adv..

[27] Belenky P., Christensen K.C., Gazzaniga F., Pletnev A.A., Brenner C. (2009). Nicotinamide Riboside and Nicotinic Acid Riboside Salvage in Fungi and Mammals. J. Biol. Chem..

[28] de Figueiredo L.F., Gossmann T.I., Ziegler M., Schuster S. (2011). Pathway analysis of NAD+ metabolism. Biochem. J..

[29] Slominski A., Wortsman J., Tobin D.J. (2004). The cutaneous serotoninergic/melatoninergic system: Securing a place under the sun. FASEB J..

[30] Slominski A.T., Zmijewski M.A., Semak I., Kim T.-K., Janjetovic Z., Slominski R.M., Zmijewski J.W. (2017). Melatonin, mitochondria, and the skin. Cell Mol. Life Sci..

[31] Kirkland J.B. (2009). Niacin Status, NAD Distribution and ADP-Ribose Metabolism. Curr. Pharm. Des..

[32] Lenglet A., Liabeuf S., Guffroy P., Fournier A., Brazier M., Massy Z.A. (2013). Use of Nicotinamide to Treat Hyperphosphatemia in Dialysis Patients. Drugs R D.

[33] Said H.M., Nexo E., Said H.M. (2018). Chapter 54-Intestinal Absorption of Water-Soluble Vitamins: Cellular and Molecular Mechanisms. Physiology of the Gastrointestinal Tract.

[34] Sadoogh-Abasian F., Evered D. (1980). Absorption of nicotinic acid and nicotinamide from rat small intestine in vitro. Biochim. Biophys. Acta Biomembr..

[35] Real A.M., Hong S., Pissios P. (2013). Nicotinamide N-Oxidation by CYP2E1 in Human Liver Microsomes. Drug Metab. Dispos..

[36] Kothawade P.B., Thomas A.B., Chitlange S.S. (2021). Novel Niacin Receptor Agonists: A Promising Strategy for the Treatment of Dyslipidemia. Mini Rev. Med. Chem..

[37] Benyó Z., Gille A., Bennett C.L., Clausen B.E., Offermanns S. (2006). Nicotinic Acid-Induced Flushing Is Mediated by Activation of Epidermal Langerhans Cells. Mol. Pharmacol..

[38] Kamanna V.S., Kashyap M.L. (2008). Mechanism of Action of Niacin. Am. J. Cardiol..

[39] Chen A.C., Martin A.J., Choy B., Fernández-Peñas P., Dalziell R.A., McKenzie C.A., Scolyer R.A., Dhillon H.M., Vardy J.L., Kricker A. (2015). A Phase 3 Randomized Trial of Nicotinamide for Skin-Cancer Chemoprevention. N. Engl. J. Med..

[40] Hwang E.S., Song S.B. (2020). Possible Adverse Effects of High-Dose Nicotinamide: Mechanisms and Safety Assessment. Biomolecules.

[41] Number 5 SV 25. Nicotinamide: An Update and Review of Safety & Differences from Niacin 2020. https://www.skintherapyletter.com/dermatology/nicotinamide-update-niacin/.

[42] Wohlrab J., Kreft D. (2014). Niacinamide-Mechanisms of Action and Its Topical Use in Dermatology. Ski. Pharmacol. Physiol..

[43] Bains P., Kaur M., Kaur J., Sharma S. (2018). Nicotinamide: Mechanism of action and indications in dermatology. Indian J. Dermatol. Venereol. Leprol..

[44] Rolfe H.M. (2014). A review of nicotinamide: Treatment of skin diseases and potential side effects. J. Cosmet. Dermatol..

[45] Gasperi V., Sibilano M., Savini I., Catani M.V. (2019). Niacin in the Central Nervous System: An Update of Biological Aspects and Clinical Applications. Int. J. Mol. Sci..

[46] Mainville L., Smilga A.-S., Fortin P.R. (2022). Effect of Nicotinamide in Skin Cancer and Actinic Keratoses Chemoprophylaxis, and Adverse Effects Related to Nicotinamide: A Systematic Review and Meta-Analysis. J. Cutan. Med. Surg..

[47] Julius U., Fischer S. (2013). Nicotinic acid as a lipid-modifying drug–A review. Atheroscler. Suppl..

[48] Prakash R., Gandotra S., Singh L.K., Das B., Lakra A. (2008). Rapid resolution of delusional parasitosis in pellagra with niacin augmentation therapy. Gen. Hosp. Psychiatry.

[49] Boden W.E., Sidhu M.S., Toth P.P. (2013). The Therapeutic Role of Niacin in Dyslipidemia Management. J. Cardiovasc. Pharmacol. Ther..

[50] Kei A., Liberopoulos E.N., Elisaf M.S. (2011). What restricts the clinical use of nicotinic acid?. Curr. Vasc. Pharmacol..

[51] Freeberg K.A., Udovich C.A.C., Martens C.R., Seals D.R., Craighead D.H. (2023). Dietary Supplementation With NAD+-Boosting Compounds in Humans: Current Knowledge and Future Directions. J. Gerontol. Ser. A.

[52] Thompson K.G., Kim N. (2020). Dietary supplements in dermatology: A review of the evidence for zinc, biotin, vitamin D, nicotinamide, and Polypodium. J. Am. Acad. Dermatol..

[53] Drago F., Ciccarese G., Cogorno L., Calvi C., Marsano L.A., Parodi A. (2017). Prevention of non-melanoma skin cancers with nicotinamide in transplant recipients: A case-control study. Eur. J. Dermatol..

[54] Murray M.F. (2003). Nicotinamide: An Oral Antimicrobial Agent with Activity against Both *Mycobacterium tuberculosis* and Human Immunodeficiency Virus. Clin. Infect. Dis..

[55] Volpi E., Lucidi P., Cruciani G., Monacchia F., Reboldi G., Brunetti P., Bolli G.B., De Feo P. (1997). Nicotinamide Counteracts Alcohol-Induced Impairment of Hepatic Protein Metabolism in Humans. J. Nutr..

[56] LeWitt P.A. (1980). The Neurotoxicity of the Rat Poison Vacor. N. Engl. J. Med..

[57] Ito T.K., Sato T., Takanashi Y., Tamannaa Z., Kitamoto T., Odagiri K., Setou M. (2021). A single oral supplementation of nicotinamide within the daily tolerable upper level increases blood NAD+ levels in healthy subjects. Transl. Med. Aging.

[58] Song Q., Zhou X., Xu K., Liu S., Zhu X., Yang J. (2023). The Safety and Antiaging Effects of Nicotinamide Mononucleotide in Human Clinical Trials: An Update. Adv. Nutr. Int. Rev. J..

[59] Reiten O.K., Wilvang M.A., Mitchell S.J., Hu Z., Fang E.F. (2021). Preclinical and clinical evidence of NAD+ precursors in health, disease, and ageing. Mech. Ageing Dev..

[60] Migaud M.E., Ziegler M., Baur J.A. (2024). Regulation of and challenges in targeting NAD+ metabolism. Nat. Rev. Mol. Cell Biol..

[61] Jackson S.P., Bartek J. (2009). The DNA-damage response in human biology and disease. Nature.

[62] Carusillo A., Mussolino C. (2020). DNA Damage: From Threat to Treatment. Cells.

[63] Caldecott K.W. (2014). DNA single-strand break repair. Exp. Cell Res..

[64] Chatterjee N., Walker G.C. (2017). Mechanisms of DNA damage, repair, and mutagenesis. Environ. Mol. Mutagen..

[65] Harrison J.C., Haber J.E. (2006). Surviving the Breakup: The DNA Damage Checkpoint. Annu. Rev. Genet..

[66] Williams A.B., Schumacher B. (2016). p53 in the DNA-Damage-Repair Process. Cold Spring Harb. Perspect. Med..

[67] Ou H.-L., Schumacher B. (2018). DNA damage responses and p53 in the aging process. Blood.

[68] Karimian A., Ahmadi Y., Yousefi B. (2016). Multiple functions of p21 in cell cycle, apoptosis and transcriptional regulation after DNA damage. DNA Repair.

[69] Nikas I.P., Paschou S.A., Ryu H.S. (2020). The Role of Nicotinamide in Cancer Chemoprevention and Therapy. Biomolecules.

[70] Chaudhuri A.R., Nussenzweig A. (2017). The multifaceted roles of PARP1 in DNA repair and chromatin remodelling. Nat. Rev. Mol. Cell Biol..

[71] Zhang H., Zha S. (2024). The dynamics and regulation of PARP1 and PARP2 in response to DNA damage and during replication. DNA Repair.

[72] Chaudhary M.R., Chaudhary S., Sharma Y., Singh T.A., Mishra A.K., Sharma S., Mehdi M.M. (2023). Aging, oxidative stress and degenerative diseases: Mechanisms, complications and emerging therapeutic strategies. Biogerontology.

[73] Hurtado-Bagès S., Knobloch G., Ladurner A.G., Buschbeck M. (2020). The taming of PARP1 and its impact on NAD+ metabolism. Mol. Metab..

[74] Chang H.-C., Guarente L. (2014). SIRT1 and other sirtuins in metabolism. Trends Endocrinol. Metab..

[75] Yang Y., Liu Y., Wang Y., Chao Y., Zhang J., Jia Y., Tie J., Hu D. (2022). Regulation of SIRT1 and Its Roles in Inflammation. Front. Immunol..

[76] Choi S.-E., Kemper J.K. (2013). Regulation of SIRT1 by MicroRNAs. Mol. Cells.

[77] Chen C., Zhou M., Ge Y., Wang X. (2020). SIRT1 and aging related signaling pathways. Mech. Ageing Dev..

[78] Avalos J.L., Bever K.M., Wolberger C. (2005). Mechanism of Sirtuin Inhibition by Nicotinamide: Altering the NAD+ Cosubstrate Specificity of a Sir2 Enzyme. Mol. Cell.

[79] Hwang E.S., Song S.B. (2017). Nicotinamide is an inhibitor of SIRT1 in vitro, but can be a stimulator in cells. Cell Mol. Life Sci..

[80] Moreno-Yruela C., Zhang D., Wei W., Bæk M., Liu W., Gao J., Danková D., Nielsen A.L., Bolding J.E., Yang L. (2022). Class I histone deacetylases (HDAC1–3) are histone lysine delactylases. Sci. Adv..

[81] Yang H., Zhang W., Pan H., Feldser H.G., Lainez E., Miller C., Leung S., Zhong Z., Zhao H., Sweitzer S. (2012). SIRT1 Activators Suppress Inflammatory Responses through Promotion of p65 Deacetylation and Inhibition of NF-κB Activity. PLoS ONE.

[82] Kim I., He Y.-Y. (2014). Ultraviolet radiation-induced non-melanoma skin cancer: Regulation of DNA damage repair and inflammation. Genes Dis..

[83] Ansary T.M., Hossain M.R., Kamiya K., Komine M., Ohtsuki M. (2021). Inflammatory Molecules Associated with Ultraviolet Radiation-Mediated Skin Aging. Int. J. Mol. Sci..

[84] Kay J., Thadhani E., Samson L., Engelward B. (2019). Inflammation-induced DNA damage, mutations and cancer. DNA Repair.

[85] Pezone A., Olivieri F., Napoli M.V., Procopio A., Avvedimento E.V., Gabrielli A. (2023). Inflammation and DNA damage: Cause, effect or both. Nat. Rev. Rheumatol..

[86] Zhao Y., Simon M., Seluanov A., Gorbunova V. (2022). DNA damage and repair in age-related inflammation. Nat. Rev. Immunol..

[87] Brzostek-Racine S., Gordon C., Van Scoy S., Reich N.C. (2011). The DNA Damage Response Induces IFN. J. Immunol..

[88] Yu H., Lin L., Zhang Z., Zhang H., Hu H. (2020). Targeting NF-κB pathway for the therapy of diseases: Mechanism and clinical study. Signal Transduct. Target. Ther..

[89] Hoesel B., Schmid J.A. (2013). The complexity of NF-κB signaling in inflammation and cancer. Mol. Cancer.

[90] Lawrence T. (2009). The Nuclear Factor NF-kappa B Pathway in Inflammation. Cold Spring Harb. Perspect. Biol..

[91] Sun S.-C. (2017). The non-canonical NF-κB pathway in immunity and inflammation. Nat. Rev. Immunol..

[92] Sun S.-C. (2010). Non-canonical NF-κB signaling pathway. Cell Res..

[93] DiDonato J.A., Mercurio F., Karin M. (2012). NF-κB and the link between inflammation and cancer. Immunol. Rev..

[94] Torres-Méndez J.K., Niño-Narvión J., Martinez-Santos P., Diarte-Añazco E.M.G., Méndez-Lara K.A., del Olmo T.V., Rotllan N., Julián M.T., Alonso N., Mauricio D. (2023). Nicotinamide Prevents Diabetic Brain Inflammation via NAD+-Dependent Deacetylation Mechanisms. Nutrients.

[95] Hou Y., Wei Y., Lautrup S., Yang B., Wang Y., Cordonnier S., Mattson M.P., Croteau D.L., Bohr V.A. (2021). NAD ^+^ supplementation reduces neuroinflammation and cell senescence in a transgenic mouse model of Alzheimer’s disease via cGAS–STING. Proc. Natl. Acad. Sci. USA.

[96] Elhassan Y.S., Kluckova K., Fletcher R.S., Schmidt M.S., Garten A., Doig C.L., Cartwright D.M., Oakey L., Burley C.V., Jenkinson N. (2019). Nicotinamide Riboside Augments the Aged Human Skeletal Muscle NAD+ Metabolome and Induces Transcriptomic and Anti-inflammatory Signatures. Cell Rep..

[97] Sahoo B.M., Banik B.K., Borah P., Jain A. (2022). Reactive Oxygen Species (ROS): Key Components in Cancer Therapies. Anti Cancer Agents Med. Chem..

[98] De Bont R., van Larebeke N. (2004). Endogenous DNA damage in humans: A review of quantitative data. Mutagenesis.

[99] Kryston T.B., Georgiev A.B., Pissis P., Georgakilas A.G. (2011). Role of oxidative stress and DNA damage in human carcinogenesis. Mutat. Res. Mol. Mech. Mutagen..

[100] Brieger K., Schiavone S., Miller F.J., Krause K.-H. (2012). Reactive oxygen species: From health to disease. Swiss Med. Wkly..

[101] Rinnerthaler M., Bischof J., Streubel M.K., Trost A., Richter K. (2015). Oxidative Stress in Aging Human Skin. Biomolecules.

[102] Sies H., Sies H., Sies H. (1985). Oxidative Stress: Introductory Remarks.

[103] Son Y., Cheong Y.-K., Kim N.-H., Chung H.-T., Kang D.G., Pae H.-O. (2011). Mitogen-Activated Protein Kinases and Reactive Oxygen Species: How Can ROS Activate MAPK Pathways?. J. Signal Transduct..

[104] Mo X., Chen X., Pan X., Lu Y., Pan G., Xie J., Pan Z., Li L., Tian H., Li Y. (2023). Protective effect of *Helianthus annuus* seed byproduct extract on ultraviolet radiation-induced injury in skin cells. Photochem. Photobiol..

[105] Lavoie H., Gagnon J., Therrien M. (2020). ERK signalling: A master regulator of cell behaviour, life and fate. Nat. Rev. Mol. Cell Biol..

[106] Boo Y.C. (2021). Mechanistic Basis and Clinical Evidence for the Applications of Nicotinamide (Niacinamide) to Control Skin Aging and Pigmentation. Antioxidants.

[107] Tan C.Y.R., Tan C.L., Chin T., Morenc M., Ho C.Y., Rovito H.A., Quek L.S., Soon A.L., Lim J.S., Dreesen O. (2021). Nicotinamide Prevents UVB- and Oxidative Stress‒Induced Photoaging in Human Primary Keratinocytes. J. Investig. Dermatol..

[108] Camillo L., Gironi L.C., Zavattaro E., Esposto E., Savoia P. (2021). Nicotinamide Attenuates UV-Induced Stress Damage in Human Primary Keratinocytes from Cancerization Fields. J. Investig. Dermatol..

[109] Chen T.-W., Wu P.-Y., Wen Y.-T., Desai T.D., Huang C.-T., Liu P.-K., Tsai R.-K. (2022). Vitamin B3 Provides Neuroprotection via Antioxidative Stress in a Rat Model of Anterior Ischemic Optic Neuropathy. Antioxidants.

[110] Cichocki F., Zhang B., Wu C.-Y., Chiu E., Day A., O’connor R.S., Yackoubov D., Simantov R., McKenna D.H., Cao Q. (2023). Nicotinamide enhances natural killer cell function and yields remissions in patients with non-Hodgkin lymphoma. Sci. Transl. Med..

[111] Doroftei B., Ilie O.-D., Cojocariu R.-O., Ciobica A., Maftei R., Grab D., Anton E., McKenna J., Dhunna N., Simionescu G. (2020). Minireview Exploring the Biological Cycle of Vitamin B3 and Its Influence on Oxidative Stress: Further Molecular and Clinical Aspects. Molecules.

[112] Li Y., Tian X., Luo J., Bao T., Wang S., Wu X. (2024). Molecular mechanisms of aging and anti-aging strategies. Cell Commun. Signal..

[113] Patel J., Baptiste B.A., Kim E., Hussain M., Croteau D.L., Bohr V.A. (2020). DNA damage and mitochondria in cancer and aging. Carcinogenesis.

[114] Shmulevich R., Krizhanovsky V. (2021). Cell Senescence, DNA Damage, and Metabolism. Antioxid. Redox Signal..

[115] Harris S.L., Levine A.J. (2005). The p53 pathway: Positive and negative feedback loops. Oncogene.

[116] Rao S.G., Jackson J.G. (2016). SASP: Tumor Suppressor or Promoter? Yes!. Trends Cancer.

[117] Di Micco R., Krizhanovsky V., Baker D., di Fagagna F.D. (2020). Cellular senescence in ageing: From mechanisms to therapeutic opportunities. Nat. Rev. Mol. Cell Biol..

[118] Lopes-Paciencia S., Saint-Germain E., Rowell M.-C., Ruiz A.F., Kalegari P., Ferbeyre G. (2019). The senescence-associated secretory phenotype and its regulation. Cytokine.

[119] Campisi J., d’Adda di Fagagna F. (2007). Cellular senescence: When bad things happen to good cells. Nat. Rev. Mol. Cell Biol..

[120] Imai S.-I., Guarente L. (2014). NAD+ and sirtuins in aging and disease. Trends Cell Biol..

[121] Kang H.T., Lee H.I., Hwang E.S. (2006). Nicotinamide extends replicative lifespan of human cells. Aging Cell.

[122] Mahajan A.S., Arikatla V.S., Thyagarajan A., Zhelay T., Sahu R.P., Kemp M.G., Spandau D.F., Travers J.B. (2021). Creatine and Nicotinamide Prevent Oxidant-Induced Senescence in Human Fibroblasts. Nutrients.

[123] Oblong J.E., Bowman A., Rovito H.A., Jarrold B.B., Sherrill J.D., Black M.R., Nelson G., Kimball A.B., Birch-Machin M.A. (2020). Metabolic dysfunction in human skin: Restoration of mitochondrial integrity and metabolic output by nicotinamide (niacinamide) in primary dermal fibroblasts from older aged donors. Aging Cell.

[124] Autier P., Doré J.-F. (2020). Ultraviolet radiation and cutaneous melanoma: A historical perspective. Melanoma Res..

[125] Yin R., Dai T., Avci P., Jorge A.E.S., de Melo W.C.M.A., Vecchio D., Huang Y., Gupta A., Hamblin M.R. (2013). Light based anti-infectives: Ultraviolet C irradiation, photodynamic therapy, blue light, and beyond. Curr. Opin. Pharmacol..

[126] Watson M., Holman D.M., Maguire-Eisen M. (2016). Ultraviolet Radiation Exposure and Its Impact on Skin Cancer Risk. Semin. Oncol. Nurs..

[127] Wolf P. (2019). Vitamin D: One more argument for broad-spectrum ultraviolet A + ultraviolet B sunscreen protection. Br. J. Dermatol..

[128] Karisma V.W., Wu W., Lei M., Liu H., Nisar M.F., Lloyd M.D., Pourzand C., Zhong J.L. (2021). UVA-Triggered Drug Release and Photo-Protection of Skin. Front. Cell Dev. Biol..

[129] Holick M.F. (2007). Vitamin D Deficiency. N. Engl. J. Med..

[130] Slominski R.M., Chen J.Y., Raman C., Slominski A.T. (2024). Photo-neuro-immuno-endocrinology: How the ultraviolet radiation regulates the body, brain, and immune system. Proc. Natl. Acad. Sci. USA.

[131] Sinha R.P., Häder D.-P. (2002). UV-induced DNA damage and repair: A review. Photochem. Photobiol. Sci..

[132] Young A.R., Claveau J., Rossi A.B. (2017). Ultraviolet radiation and the skin: Photobiology and sunscreen photoprotection. J. Am. Acad. Dermatol..

[133] Brenner M., Hearing V.J. (2008). The Protective Role of Melanin Against UV Damage in Human Skin. Photochem. Photobiol..

[134] Matsumura Y., Ananthaswamy H.N. (2002). Short-term and long-term cellular and molecular events following UV irradiation of skin: Implications for molecular medicine. Expert Rev. Mol. Med..

[135] Kammeyer A., Luiten R.M. (2015). Oxidation events and skin aging. Ageing Res. Rev..

[136] D’Orazio J., Jarrett S., Amaro-Ortiz A., Scott T. (2013). UV Radiation and the Skin. Int. J. Mol. Sci..

[137] Matsumura Y., Ananthaswamy H.N. (2004). Toxic effects of ultraviolet radiation on the skin. Toxicol. Appl. Pharmacol..

[138] Schwarz T. (2005). Mechanisms of UV-induced immunosuppression. Keio J. Med..

[139] Slominski A.T., Zmijewski M.A., Plonka P.M., Szaflarski J.P., Paus R. (2018). How UV Light Touches the Brain and Endocrine System Through Skin, and Why. Endocrinology.

[140] Damian D.L. (2017). Nicotinamide for skin cancer chemoprevention. Australas. J. Dermatol..

[141] Pellacani G., Lim H.W., Stockfleth E., Sibaud V., Brugués A.O., Aroman M.S. (2024). Photoprotection: Current developments and controversies. J. Eur. Acad. Dermatol. Venereol..

[142] Yiasemides E., Sivapirabu G., Halliday G.M., Park J., Damian D.L. (2008). Oral nicotinamide protects against ultraviolet radiation-induced immunosuppression in humans. Carcinogenesis.

[143] Damian D.L., Patterson C.R.S., Stapelberg M., Park J., Barnetson R.S.C., Halliday G.M. (2008). UV Radiation-Induced Immunosuppression Is Greater in Men and Prevented by Topical Nicotinamide. J. Investig. Dermatol..

[144] Camillo L., Gironi L.C., Esposto E., Zavattaro E., Savoia P. (2022). Nicotinamide and calcipotriol counteract UVB-induced photoaging on primary human dermal fibroblasts. J. Photochem. Photobiol..

[145] Bierman J.C., Laughlin T., Tamura M., Hulette B.C., Mack C.E., Sherrill J.D., Tan C.Y.R., Morenc M., Bellanger S., Oblong J.E. (2020). Niacinamide mitigates SASP-related inflammation induced by environmental stressors in human epidermal keratinocytes and skin. Int. J. Cosmet. Sci..

[146] Park J., Halliday G.M., Surjana D., Damian D.L. (2010). Nicotinamide Prevents Ultraviolet Radiation-induced Cellular Energy Loss. Photochem. Photobiol..

[147] Surjana D., Halliday G.M., Damian D.L. (2013). Nicotinamide enhances repair of ultraviolet radiation-induced DNA damage in human keratinocytes and ex vivo skin. Carcinogenesis.

[148] Monfrecola G., Gaudiello F., Cirillo T., Fabbrocini G., Balato A., Lembo S. (2013). Nicotinamide downregulates gene expression of interleukin-6, interleukin-10, monocyte chemoattractant protein-1, and tumour necrosis factor-α gene expression in HaCaT keratinocytes after ultraviolet B irradiation. Clin. Exp. Dermatol..

[149] Chhabra G., Garvey D.R., Singh C.K., Mintie C.A., Ahmad N. (2018). Effects and Mechanism of Nicotinamide Against UVA- and/or UVB-mediated DNA Damages in Normal Melanocytes. Photochem. Photobiol..

[150] Fisher G.J., Kang S., Varani J., Bata-Csorgo Z., Wan Y., Datta S., Voorhees J.J. (2002). Mechanisms of Photoaging and Chronological Skin Aging. Arch. Dermatol..

[151] Najafabadi A.H., Soheilifar M.H., Masoudi-Khoram N. (2024). Exosomes in skin photoaging: Biological functions and therapeutic opportunity. Cell Commun. Signal..

[152] Mineroff J., Nguyen J.K., Jagdeo J. (2024). Mineroff J, Nguyen JK, Jagdeo J. The Importance of Photoaging Prevention in All Skin Types: An Update on Current Advancements. J. Drugs Dermatol..

[153] Bissett D.L., Miyamoto K., Sun P., Li J., Berge C.A. (2004). Topical niacinamide reduces yellowing, wrinkling, red blotchiness, and hyperpigmented spots in aging facial skin. Int. J. Cosmet. Sci..

[154] Vergilio M.M., Leonardi G.R. (2024). Topical Formulation with Niacinamide Combined with 5 MHz Ultrasound for Improving Skin Ageing: A Double-blind, Randomised, Placebo-controlled Clinical Study. Curr. Med. Chem..

[155] Bogdanowicz P., Bensadoun P., Noizet M., Béganton B., Philippe A., Alvarez-Georges S., Doat G., Tourette A., Bessou-Touya S., Lemaitre J.-M. (2024). Senomorphic activity of a combination of niacinamide and hyaluronic acid: Correlation with clinical improvement of skin aging. Sci. Rep..

[156] Allen N.C., Martin A.J., Snaidr V.A., Eggins R., Chong A.H., Fernandéz-Peñas P., Gin D., Sidhu S., Paddon V.L., Banney L.A. (2023). Nicotinamide for Skin-Cancer Chemoprevention in Transplant Recipients. N. Engl. J. Med..

[157] Martins I.M.d.C., Miot H.A., Miola A.C. (2023). Effectiveness and safety of 5% nicotinamide cream following cryosurgery in skin field cancerization: A randomized, double-blind, placebo-controlled clinical trial. Int. J. Dermatol..

[158] Veronese F., Seoni S., Tarantino V., Buttafava M., Airoldi C., Meiburger K.M., Zavattaro E., Savoia P. (2022). AKASI and Near-Infrared Spectroscopy in the combined effectiveness evaluation of an actinic keratoses preventive product in immunocompetent and immunocompromised patients. Front. Med..

[159] Shalita A.R., Smith J.G., Parish L.C., Sofman M.S., Chalker D.K. (1995). Topical Nicotinamide Compared with Clindamycin Gel in the Treatment of Inelammatory Acne Vulgaris. Int. J. Dermatol..

[160] Tempark T., Shem A., Lueangarun S. (2024). Efficacy of ceramides and niacinamide-containing moisturizer versus hydrophilic cream in combination with topical anti-acne treatment in mild to moderate acne vulgaris: A split face, double-blinded, randomized controlled trial. J. Cosmet. Dermatol..

[161] Khodaeiani E., Fouladi R.F., Amirnia M., Saeidi M., Karimi E.R. (2013). Topical 4% nicotinamide vs. 1% clindamycin in moderate inflammatory acne vulgaris. Int. J. Dermatol..

[162] Shahmoradi Z., Iraji F., Siadat A.H., Ghorbaini A. (2013). Comparison of topical 5% nicotinamid gel versus 2% clindamycin gel in the treatment of the mild-moderate acne vulgaris: A double-blinded randomized clinical trial. J. Res. Med. Sci..

[163] Emanuele E., Bertona M., Altabas, Alessandrini G., Altabas K., Altabas V. (2012). Anti-inflammatory effects of a topical preparation containing nicotinamide, retinol, and 7-dehydrocholesterol in patients with acne: A gene expression study. Clin. Cosmet. Investig. Dermatol..

[164] De Lucas R., Martínez H., Nieto C., Ruiz-Alonso C., Bermejo R., Carrón N., Garcia-Segura S., Gonzalez-Torres P., Palacios-Martínez D., Guerra-Tapia A. (2024). New clinical approach in facial mild–moderate acne: Re-stabilization of skin microbiota balance with a topical biotechnological phytocomplex. J. Cosmet. Dermatol..

[165] Morganti P., Berardesca E., Guarneri B., Guarneri F., Fabrizi G., Palombo P., Palombo M. (2011). Topical clindamycin 1% vs. linoleic acid-rich phosphatidylcholine and nicotinamide 4% in the treatment of acne: A multicentre-randomized trial. Int. J. Cosmet. Sci..

[166] Li W., Yu Q., Shen Z., Zhang L., Zhang W., Li C. (2022). Efficacy and safety of a cream containing octyl salicylic acid, salicylic acid, linoleic acid, nicotinamide, and piroctone olamine combined with 5% benzoyl peroxide in the treatment of acne vulgaris: A randomized controlled study. Chin. Med. J..

[167] Niren N.M., Torok H.M. (2006). The Nicomide Improvement in Clinical Outcomes Study (NICOS): Results of an 8-week trial. Cutis.

[168] Cannizzaro M.V., Dattola A., Garofalo V., Del Duca E., Bianchi L. (2018). Reducing the oral isotretinoin skin side effects: Efficacy of 8% omega-ceramides, hydrophilic sugars, 5% niacinamide cream compound in acne patients. Ital. J. Dermatol. Venereol..

[169] Honl B.A., Elston D.M. (1998). Autoimmune bullous eruption localized to a breast reconstruction site: Response to niacinamide. Cutis.

[170] Berk M.A., Lorincz A.L. (1986). The Treatment of Bullous Pemphigoid With Tetracycline and Niacinamide. Arch. Dermatol..

[171] Kolbach D.N., Remme J.J., Bos W.H., Jonkman M.F., Jong M.C., Pas H.H., Meer J.B. (1995). Bullous pemphigoid successfully controlled by tetracycline and nicotinamide. Br. J. Dermatol..

[172] Hornschuh B., Hamm H., Wever S., Hashimoto T., Schröder U., Bröcker E.-B., Zillikens D. (1997). Treatment of 16 patients with bullous pemphigoid with oral tetracycline and niacinamide and topical clobetasol. J. Am. Acad. Dermatol..

[173] Fivenson D.P., Kimbrough T.L. (1997). Lichen planus pemphigoides: Combination therapy with tetracycline and nicotinamide. J. Am. Acad. Dermatol..

[174] Hughes A.P., Callen J.P. (2001). Epidermolysis Bullosa Acquisita Responsive to Dapsone Therapy. J. Cutan. Med. Surg..

[175] Goon A.T., Tan S.H., Khoo L.S., Tan T. (2000). Tetracycline and nicotinamide for the treatment of bullous pemphigoid: Our experience in Singapore. Singap. Med. J..

[176] Kalinska-Bienias A., Kowalczyk E., Jagielski P., Kowalewski C., Wozniak K. (2018). Tetracycline, nicotinamide, and lesionally administered clobetasol as a therapeutic option to prednisone in patients with bullous pemphigoid: A comparative, retrospective analysis of 106 patients with long-term follow-up. Int. J. Dermatol..

[177] El-Heis S., Crozier S.R., Robinson S.M., Harvey N.C., Cooper C., Inskip H.M., Godfrey K., Southampton Women’s Survey Study Group (2016). Higher maternal serum concentrations of nicotinamide and related metabolites in late pregnancy are associated with a lower risk of offspring atopic eczema at age 12 months. Clin. Exp. Allergy.

[178] Tanno O., Ota Y., Kitamura N., Katsube T., Inoue S. (2000). Nicotinamide increases biosynthesis of ceramides as well as other stratum corneum lipids to improve the epidermal permeability barrier. Br. J. Dermatol..

[179] Xu A., Song X., Pan W., Wallin B., Kivlin R., Lu S., Cao C., Bi Z., Wan Y. (1998). Nicotinamide attenuates aquaporin 3 overexpression induced by retinoic acid through inhibition of EGFR/ERK in cultured human skin keratinocytes. Int. J. Mol. Med..

[180] Soma Y., Kashima M., Imaizumi A., Takahama H., Kawakami T., Mizoguchi M. (2004). Moisturizing effects of topical nicotinamide on atopic dry skin. Int. J. Dermatol..

[181] Zhu J., Wang J., Wang S. (2023). A single-center, randomized, controlled study on the efficacy of niacinamide-containing body emollients combined with cleansing gel in the treatment of mild atopic dermatitis. Ski. Res. Technol..

[182] Yu K., Wang Y., Wan T., Zhai Y., Cao S., Ruan W., Wu C., Xu Y. (2017). Tacrolimus nanoparticles based on chitosan combined with nicotinamide: Enhancing percutaneous delivery and treatment efficacy for atopic dermatitis and reducing dose. Int. J. Nanomed..

[183] Wozniacka A., Wieczorkowska M., Gebicki J., Sysa-Jedrzejowska A. (2005). Topical application of 1-methylnicotinamide in the treatment of rosacea: A pilot study. Clin. Exp. Dermatol..

[184] Torregrosa A., Ochoa-Andrade A.T., Parente M.E., Vidarte A., Guarinoni G., Savio E. (2020). Development of an emulgel for the treatment of rosacea using quality by design approach. Drug Dev. Ind. Pharm..

[185] Draelos Z.D., Ertel K., Berge C. (2005). Niacinamide-containing facial moisturizer improves skin barrier and benefits subjects with rosacea. Cutis.

[186] Fowler J.F., Woolery-Lloyd H., Waldorf H., Saini R. (2010). Innovations in natural ingredients and their use in skin care. J. Drugs Dermatol..

[187] Berardesca E., Bonfigli A., Cartigliani C., Kerob D., Tan J. (2023). A Randomized, Controlled Clinical Trial of a Dermocosmetic Containing Vichy Volcanic Mineralizing Water and Probiotic Fractions in Subjects with Rosacea Associated with Erythema and Sensitive Skin and Wearing Protective Masks. Clin. Cosmet. Investig. Dermatol..

[188] Levine D., Even-Chen Z., Lipets I., Pritulo O.A., Svyatenko T.V., Andrashko Y., Lebwohl M., Gottlieb A. (2010). Pilot, multicenter, double-blind, randomized placebo-controlled bilateral comparative study of a combination of calcipotriene and nicotinamide for the treatment of psoriasis. J. Am. Acad. Dermatol..

[189] El-Khalawany M., Nouh A.H., Kadah A.S., Elsheikh M., Said M. (2022). Evaluation of safety and efficacy of topical 4% nicotinamide in treatment of psoriasis; among a representative sample of Egyptians (an analytical observational study). Dermatol. Ther..

[190] Cao A.-P., Wang Y.-Y., Shen Y.-Y., Liu Y.-H., Liu J.-Y., Wang Y., Guo Y., Wang R.-B., Xie B.-Y., Pan X. (2024). Nicotinamide Suppresses Hyperactivation of Dendritic Cells to Control Autoimmune Disease through PARP Dependent Signaling. Nutrients.

[191] Kimball A., Kaczvinsky J., Li J., Robinson L., Matts P., Berge C., Miyamoto K., Bissett D. (2009). Reduction in the appearance of facial hyperpigmentation after use of moisturizers with a combination of topical niacinamide and *N* -acetyl glucosamine: Results of a randomized, double-blind, vehicle-controlled trial. Br. J. Dermatol..

[192] Crocco E.I., Torloni L., Fernandes P.B., de Campos M.E.B.B., Gonzaga M., Silva F.C., Nasario J.P.S., Guerra L.O., Csipak A.R., Castilho V.C. (2024). Combination of 5% cysteamine and 4% nicotinamide in melasma: Efficacy, tolerability, and safety. J. Cosmet. Dermatol..

[193] Navarrete-Solís J., Castanedo-Cázares J.P., Torres-Álvarez B., Oros-Ovalle C., Fuentes-Ahumada C., González F.J., Martínez-Ramírez J.D., Moncada B. (2011). A Double-Blind, Randomized Clinical Trial of Niacinamide 4% versus Hydroquinone 4% in the Treatment of Melasma. Dermatol. Res. Pract..

[194] Campuzano-García A.E., Torres-Alvarez B., Hernández-Blanco D., Fuentes-Ahumada C., Cortés-García J.D., Castanedo-Cázares J.P. (2019). DNA Methyltransferases in Malar Melasma and Their Modification by Sunscreen in Combination with 4% Niacinamide, 0.05% Retinoic Acid, or Placebo. BioMed Res. Int..

[195] Liang Y., Li M., Tang Y., Yang J., Wang J., Zhu Y., Liang H., Lin Q., Cheng Y., Yang X. (2023). Temperature-sensitive hydrogel dressing loaded with nicotinamide mononucleotide accelerating wound healing in diabetic mice. Biomed. Pharmacother..

[196] Esfahani S.A., Khoshneviszadeh M., Namazi M.R., Noorafshan A., Geramizadeh B., Nadimi E., Razavipour S.T. (2015). Topical Nicotinamide Improves Tissue Regeneration in Excisional Full-Thickness Skin Wounds: A Stereological and Pathological Study. Trauma Mon..

[197] Zhang C., Shao Q., Zhang Y., Liu W., Kang J., Jin Z., Huang N., Ning B. (2024). Therapeutic application of nicotinamide: As a potential target for inhibiting fibrotic scar formation following spinal cord injury. CNS Neurosci. Ther..

